# Evidence for ABL Amplification in Multiple Myeloma and Therapeutic Implications

**DOI:** 10.1155/2022/4112016

**Published:** 2022-03-15

**Authors:** He Huang, Shuping Zhou, Hongdou Lin, Wenjian Guo, Ying Lin, Ronxin Yao, Licai He, Kang Yu, Qian Li

**Affiliations:** ^1^Department of Hematology, The Second Affiliated Hospital and Yuying Children's Hospital of Wenzhou Medical University, Wenzhou, Zhejiang, China; ^2^Department of Hematology, Ningbo Yinzhou Second Hospital, Ningbo, Zhejiang, China; ^3^Key Laboratory of Laboratory Medicine, Ministry of Education, School of Laboratory Medicine and Life Sciences, Wenzhou Medical University, Wenzhou, Zhejiang, China; ^4^Department of Hematology, The First Affiliated Hospital of Wenzhou Medical University, Wenzhou, Zhejiang, China; ^5^Department of Clinical Laboratory, The Second Affiliated Hospital and Yuying Children's Hospital of Wenzhou Medical University, Wenzhou, Zhejiang, China

## Abstract

**Background:**

Cytogenetic abnormalities are considered initiating events in the pathogenesis of multiple myeloma (MM) and are assumed to be of clinical significance.

**Methods:**

Fluorescence in situ hybridization (FISH) was used to analyze chromosomal architecture in 101 patients with MM. We evaluated overall patient survival and assessed the cytotoxicity of imatinib against MM cells using a CCK8 assay.

**Results:**

ABL gene amplification was detected in 67 patients (66.3%). However, ABL gene amplification was not associated with clinical features, cytogenetic abnormalities (c-Myc amplification, IGH rearrangement, RB1 deletion, p53 deletion, or 1q21 amplification), or overall survival. ABL amplification in MM cell lines (LP-1 and U266) was revealed by FISH. Furthermore, the ABL protein was easily detectable in MM cell lines and some tumor cells by western blotting. A CCK8 assay indicated limited cytotoxicity of imatinib against MM cells.

**Conclusions:**

Our study firstly discussed ABL gene amplification was prevalent in MM cells, and we believe that the ABL gene would potentially be a useful target in the treatment of combination strategy for MM with ABL amplification in the future.

## 1. Introduction 

Multiple myeloma (MM) is one of the most common hematological tumors and is characterized by the malignant proliferation of plasma cells. Despite encouraging therapeutic advances, the disease remains incurable because of complex genomic alterations, low sensitivity of MM cells to chemotherapy in the bone marrow microenvironment, and the emergence of drug resistance [1]. Accumulating evidence suggests that at the time of diagnosis, genotypic changes can be detected in approximately 60% of patients by conventional chromosome analysis and in up to 90% of patients by fluorescence in situ hybridization (FISH). Some genetic abnormalities have been identified and include rearrangement of the 14q32 (IGH) locus/c-Myc/cyclin D1/FGFR3/cyclin D3, monoallelic deletions of chromosome 13, mutations of the K-Ras and N-Ras genes, and p53 monoallelic loss [2]. Using DNA microarrays, the gene expression profiles of malignant plasma cells from nine patients with MM and eight MM cell lines were compared with those of highly purified nonmalignant plasma cells from eight individuals following in vitro differentiation of peripheral blood B cells. Overall, 250 genes were significantly up-regulated, and 159 were down-regulated in malignant plasma cells compared with normal cells. Some of these differentially expressed genes, including ABL, are over-expressed in MM cells and code for enzymes that could be targeted with drugs [3].

ABL is activated by chromosomal translocations in kinds of hematopoietic malignancies, particularly chronic myeloid leukemia (CML) characterized in almost all cases by the t (9; 22) (q34; q11) translocation. In addition, ABL fusion proteins are inseparable from tyrosine kinase activity, so small-molecule inhibitors (e.g., imatinib, also known as STI571 or Gleevec) can achieve therapeutic outcomes by targeting kinase activity. ABL is localized both in the nucleus and the cytoplasm. DNA damage and genomic instability lead to the high expression of ABL in the MM nucleus, and the disruption of the ABL-YAP1-p73 axis damages most nuclear tumor suppressor functions. Low YAP1 levels prevent nuclear ABL-induced apoptosis following endogenous DNA damage, thus representing a new synthetic-lethal strategy to selectively target cancer cells [4, 5].

FISH is versatile and expandable that can greatly facilitate research and diagnostics. Here, we used FISH to perform chromosomal profiling and undertake an analysis of ABL amplification in MM bone marrow samples and cell lines. The ABL gene was frequently amplified in MM cells, often threefold or fourfold. Compared with CML cells bearing the BCR-ABL fusion gene, imatinib monotherapy had limited antitumor effects on MM cells with ABL amplification as shown by cell proliferation assays. ABL has been identified as a selective target for synthetic-lethal strategies in MM [4]. Thus, information on ABL regulatory mechanisms is being mined to suggest potential therapeutic strategies against hematopoietic malignancies [6, 7], especially as part of combination therapies.

## 2. Materials and Methods

### 2.1. Patients

We screened the databases of the clinical cytogenetics laboratories at the Second Affiliated Hospital and Yuying Children's Hospital of Wenzhou Medical University from June 2009 to July 2018. A total of 101 patients with MM, including 11 patients with relapsed MM, were enrolled based on the International Myeloma Working Group criteria. Baseline data included sex, age, M protein subtype in serum or urine, Durie-Salmon (DS) stage, serum levels of creatinine, albumin, *β*_2_-microglobulin (*β*_2-_MG), and lactate dehydrogenase (LDH), and bone marrow karyotype of the 101 patients, and only 82 cases received at least one type chemotherapy regimen as follows: vincristine + adriamycin/doxorubicin + dexamethasone (42 cases), bortezomib + dexamethasone (36), lenalidomide + dexamethasone (11), lenalidomide + bortezomib + dexamethasone (4), ixazomib + dexamethasone (1), lenalidomide + ixazomib + dexamethasone (3), and thalidomide + dexamethasone (8). None of them had received autologous or allogeneic stem cell transplantation or immunotherapy. All patients provided written informed consent for use of their samples. The study was approved by the ethics committees of the Second Affiliated Hospital and Yuying Children's Hospital of Wenzhou Medical University.

### 2.2. Cell Lines, Proteins, and Reagents

K562, NCI–H929, LP-1, and U266 cell lines were obtained from the ATCC (Manassas, VA, USA). Cells were cultured in RPMI 1640 medium (Sigma-Aldrich, St. Louis, MO, USA) supplemented with 10% (v/v) heat-inactivated fetal bovine serum (Gibco BRL, Gaithersburg, MD, USA) at 37°C under a humidified atmosphere containing 5% CO_2_. Protein extracts from RPMI8226, NB4, U937, Kasumi-1, 293T, HCC1937, PC3, and OVCAR-3 cells were kind gifts from Professor Yingli Wu (Department of Pathophysiology, Key Laboratory of Cell Differentiation and Apoptosis, Chinese Ministry of Education Shanghai Jiao-Tong University School of Medicine). Imatinib (STI571) was purchased from Selleck Chemicals (Houston, TX, USA). A 1-mM stock solution was prepared in dimethyl sulfoxide and stored at −20°C.

### 2.3. Conventional Karyotyping

Bone marrow cells from patients with MM were cultivated for 24 to 48 hours without mitogen stimulation and harvested for chromosomal examination using standard techniques. At least 20 metaphases with R-banding using Giemsa stain were examined. The International System for Human Cytogenetic Nomenclature (2009) was used to describe chromosomal abnormalities.

### 2.4. Fluorescence In Situ Hybridization

We used commercially available probes targeting c-Myc (8q24), D13S319/RB1/(13q14), p53/1q21 (17p13.1/1q21), IGH (14q32), CSP17/CDKN2A (p16) (9p21), and BCR/ABL (22q11/9q34) (GP Medical, Beijing, China) to analyze 101 specimens by inter/metaphase FISH. Interphase signals were evaluated in 200 nuclei. The expression of two green and two orange signals was considered normal. Images were captured using a Nikon 80-A1 fluorescent microscope (eyepiece: NIKON Japan 10×; objective: NIKON Japan 100×; NIKON, Tokyo, Japan) and analyzed using image analysis software AI (Gene Company, Beijing, China). The color filters included SP100v2 DAPI c126877, 61000v2 D/F/R c126876, and FITC ex465-495 DM505 BA515-555. Images were captured using a camera from Bino Photo.

### 2.5. Single-Nucleotide Polymorphism Array

DNA was extracted from bone marrow aspirates collected in tubes containing ethylenediaminetetraacetic acid. Genome-wide hybridization was performed using an Affymetrix 750k SNP microarray. The procedure was carried out strictly according to the Affymetrix standard operating procedure. The assay can detect copy number variation and loss of heterozygosity at genome scale. If all quality control standards were acceptable, data analysis was carried out using Affymetrix ChAs software. The analysis was carried out using normal control standards from the Affymetrix Normal Population Gene Database.

### 2.6. Cell Proliferation Assay

Cells were seeded in 96-well plates and incubated with various concentrations of drugs in triplicate for 48 hours. Cell proliferation was assayed using a Cell Counting Kit-8 (CCK8) (Dojindo Laboratories, Kumamoto, Japan) according to the manufacturer's instructions. Each experiment was conducted in triplicate, and experiments were repeated three times.

### 2.7. Western Blot

Cells were harvested, washed with PBS, and lysed with lysis buffer (62.5 mM Tris-HCl, pH 6.8, 100 mM DTT, 2% SDS, 10% glycerol). Cell lysates were centrifuged at 20,000 g for 10 minutes at 4°C, and proteins in the supernatants were quantified. Protein extracts were equally loaded to 6% to 15% SDS–PAGE gels, electrophoresed, and transferred to nitrocellulose membrane. After blocking with 5% nonfat milk in PBS for 2 hours at room temperature, the membranes were incubated with antibodies against c-ABL that was purchased from Santa Cruz Biotechnology (Santa Cruz, CA) overnight at 4°C followed by horseradish peroxidase (HRP)-conjugated secondary antibodies for 1 hour at room temperature. Signals were detected using a chemiluminescence phototope-HRP kit (Millipore, Burlington, MA, USA) according to the manufacturer's instructions. Blotting using a monoclonal antibody against *α*-tubulin (Cell Signaling Technology, Danvers, MA, USA) was used as an internal control.

### 2.8. Statistical Analysis

Depending on the distribution, continuous data were presented as medians (interquartile ranges) or as means ± standard deviations. Categorical data were presented as counts or proportions. Differences between groups were assessed using *χ*^2^ tests or Fisher's exact tests for categorical data and using nonparametric Wilcoxon rank-sum tests or Student's *t*-tests for continuous data. Univariate and multivariate logistic regression analyses were used to assess the relationship between ABL amplification and normal ABL groups. Statistical analyses were performed using Empower (R) (http://www.empowerstats.com, X & Y Solutions, Boston, MA, USA) and R (http://www.R-project.org). Survival curves were constructed using the Kaplan-Meier method and compared using the log-rank (Mantel–Cox) test. A two-tailed value of *P* < 0.05 was considered statistically significant.

## 3. Results

### 3.1. ABL Amplification Is Frequent in Patients with MM

A total of 101 patients with MM (including 11 patients with relapsed MM) were enrolled in the study. We assessed the cytogenetic characteristics of each patient's bone marrow cells. Patient baseline demographic and clinical features are summarized in Table 1. Among the MM patients, 67 patients (66.3%) had cytogenetic abnormalities with ABL amplification identified by FISH. No significant differences were observed between patients with ABL amplification and those with normal ABL gene in terms of sex, age, M protein, DS stage, LDH, creatinine, albumin, *β*_2_-MG, or karyotype. The frequencies of c-Myc amplification, IGH rearrangement, p53 deletion, and 1q21 amplification were higher in patients with ABL amplification than in those with the normal ABL group. The frequency of D13S319/RB1 deletion was lower in patients with ABL amplification than in those with normal ABL gene (56.7% vs. 64.8%). However, differences in cytogenetic abnormalities (Table 1) were not statistically significant (*P* > 0.05). In contrast, significant differences in karyotype were observed between the two groups. Hyperdiploidy, reflecting better prognosis [8], was more frequent in patients with ABL amplification (32.8%, 22/67) than in patients with normal ABL gene (5.9%, 2/34). Normal karyotypes were present in 65.7% (44/67) of patients with ABL amplification and 76.5% (26/34) of patients with normal ABL gene. Hypodiploidy, reflecting poor prognosis [9], was detected in 17.6% (6/34) of patients with the normal ABL group and no patients with ABL amplification. Only one sample exhibited polyploidy; this sample showed ABL amplification, c-Myc amplification, IGH rearrangement, p53 deletion, and 1q21 amplification. Thus, ABL amplification was more frequent in MM cells with hyperdiploidy.

**Figure 1:**
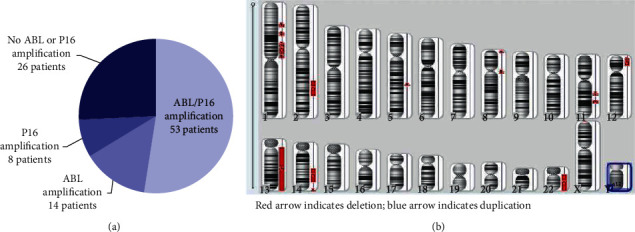
The relationship of abnormalities of chromosome 9 and ABL amplification. (a). Distribution of ABL and p16 amplification in patients with multiple myeloma (MM). (b). Single-nucleotide polymorphism (SNP) array analysis of bone marrow from a patient with MM.

Because ABL is located on chromosome 9, which is frequently duplicated as a numerical chromosomal abnormality in MM, we further examined CDKN2A p16 (located at 9p21) to exclude the possibility of trisomy chromosome 9. And p16 amplification was inconsistent with ABL amplification (Figure 1(a)). Moreover, we tested several samples by SNP array after CD138^+^ cell sorting and found that samples with ABL amplification had normal chromosome 9.  Figure 1(b) shows SNP array results for one patient who had ABL amplification but no increase on chromosome 9. Thus, we concluded that ABL amplification was not caused by chromosome 9 increase.

**Figure 2:**
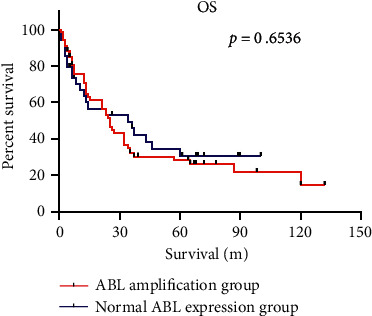
Survival analyses of MM patients. Kaplan-Meier survival curves showing relative survival of patients with ABL amplification (*n* = 63) and those with normal ABL gene (*n* = 31).

We further analyzed differences in overall survival (OS) between MM patients with ABL amplification and those with normal ABL gene. Seven patients were lost to follow-up (four patients with ABL amplification and three with normal ABL gene). The median survival of patients with ABL amplification and those with normal ABL gene was 25 months and 34 months, respectively. This difference was not statistically significant (*P* > 0.05) (Figure 2).

### 3.2. Chromosomal Characteristics of MM Cell Lines

**Figure 3:**
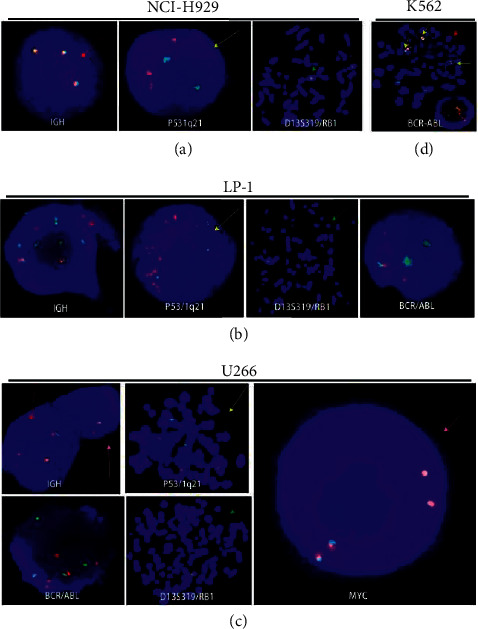
Copy number abnormalities were detected by FISH in MM cell lines. GLP IGH dual-color breakpoint probe (located at 14q32), P53/1q21 probe (located at 17p13.1/1q21), D13S319/RB1 probe (located at 13q14), GLP C-MYC dual-color breakpoint probe (located at 8q24), and GLP BCR-ABL dual-color fusion probe (located at 22q11/9q34). (a). In interphase FISH of NCI–H929 cells, the image shows three fusion signals indicating IGH amplification (red arrow), as well as two green signals and three orange signals signifying normal p53 performance and 1q21 amplification (yellow arrow). In metaphase FISH, the image shows three green and three orange signals indicating D13S319 and RB1 amplification (potential transformation of chromosome 13 into derivative chromosome 13) (green arrow). (b). LP-1: In interphase FISH, the image shows two fusions, six green signals, and two orange signals indicating IGH rearrangement and amplification (red arrow) as well as three green signals and ten orange signals indicating p53 and 1q21 amplification (yellow arrow). The image shows three green and three orange signals indicating BCR and ABL amplification (blue arrow). In metaphase FISH, the image shows two green and two orange signals on two derivative chromosome 13 (green arrow). (c). In interphase FISH of diploid U266 cells, the image shows one fusion and one orange signal indicating IGH rearrangement and IGH variable region deletion (red arrow). The image displays four green and four orange signals indicating BCR and ABL amplification (blue arrow) as well as four fusion signals indicating c-Myc amplification (pink arrow). In tetraploid cells, two fusion and two orange signals indicate IGH rearrangement and IGH variable region deletion (red arrow). In metaphase FISH, the image shows two green signals and six orange signals indicating p53 deletion and 1q21 amplification (yellow arrow) as well as two green and two orange signals indicating D13S319 and RB1 deletion (green arrow). (d). In metaphase FISH of K562 cells, the image shows two isodicentric Philadelphia chromosomes (yellow arrow), another fusion signal (yellow arrow), three orange ABL (red arrow), and two BCR performances. In interphase FISH, the image shows multiple fusion genes, three orange ABL, and two BCR performances.

To further assess the performance of FISH probes targeting small genomic loci in MM cell lines, we assessed the human MM cell lines LP-1, U266, and NCI–H929 (characterized by hyperdiploidy with 61–69 chromosomes and a variety of structural abnormalities) and the CML cell line K562 (characterized by hyperdiploidy and multiple BCR-ABL fusion gene amplifications). The GLP IGH dual-color breakpoint probe (located at 14q32), p53/1q21 probe (located at 17p13.1/1q21), D13S319/RB1 probe (located at 13q14), GLP c-Myc dual-color breakpoint probe (located at 8q24), and GLP BCR-ABL dual-color fusion probe (located at 22q11/9q34) were detected by FISH in MM cell lines. As shown in Table 2, we observed ABL amplification in LP-1 cells (three or four orange signals for the ABL gene) and U266 cells (four orange signals for the ABL gene). By contrast, NCI–H929 cells had normal ABL gene. Visualization of chromosomal territories using FISH chromosome-spotting probes revealed genes indicating poor prognosis in agreement with Hi-C measurements. In NCI–H929 cells, c-Myc, IGH, D13S319/RB1, and 1q21 appeared to have undergone triple amplification. In LP-1 cells, c-Myc was amplified sixfold, 1q21 was amplified eightfold–tenfold, and IGH was rearranged. In U266 cells, c-Myc was amplified 4 fold, 1q21 was amplified 6 fold, and the variable region of IGH, p53, and D13S319/RB1 were deleted. As shown in Figures 3(a)–3(c), (c)-Myc amplification, IGH rearrangement, p53 deletion, and 1q21 amplification were confirmed by metaphase or interphase FISH in MM cell lines. In addition, D13S319/RB1 showed different chromosomal abnormalities (amplification in NCI–H929 cells; normal in LP-1 cells; loss in U266 cells). In K562 cells, multiple BCR-ABL fusion gene amplifications were detected by FISH as a positive control (Figure 3(d)).

### 3.3. ABL Protein Expression in MM Cell Lines

**Figure 4:**
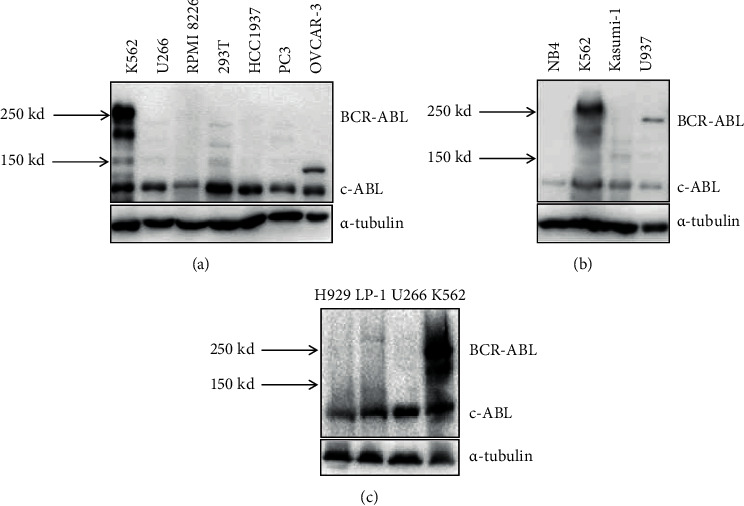
ABL protein expression in MM cells. ABL protein expression in tumor cells is measured by western blotting.

ABL genes are found in all metazoans. ABL fusion genes can transform human fibroblasts in cultures, and enhanced ABL signaling may contribute to epithelial cell malignancies and to invasive growth of breast cancer cells [10–12]. We next examined ABL protein expression in both hematologic cancer cells and solid tumors. ABL was relatively easily detected in most cells (Figure 4). Expression of c-ABL could be identified in NCI–H929, LP-1, U266, and RPMI 8226 cells; none of these cells have BCR-ABL fusion proteins. As described above, FISH was used to analyze ABL amplification in the nuclei of MM cell lines. NCI–H929 cells did not show ABL amplification. Because c-ABL localizes both the nucleus and the cytoplasm, ABL protein levels are not predictive of FISH performance.

### 3.4. Cytotoxicity of the ABL Inhibitor Imatinib against MM Cells

**Figure 5:**
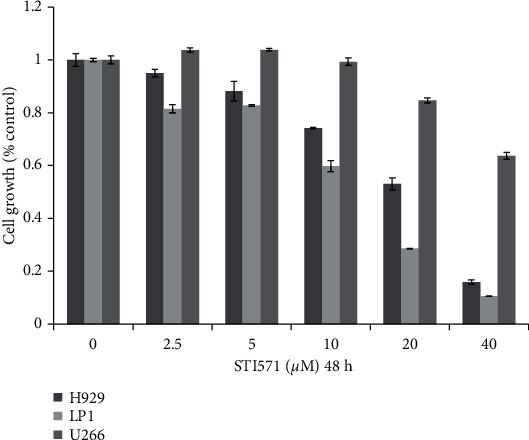
Effect of imatinib on the proliferation of MM cells. MM cell lines (NCI–H929, LP-1, and U266) are cultured in the presence of imatinib (STI571) for 48 hours, and proliferation is assayed using a CCK8 assay. All values represent means ± standard deviations of three independent experiments, and each performed in triplicate.

The concentration of imatinib necessary to obtain 50% inhibition in MM cell proliferation (IC_50_) was high (>10 *μ*M) (Figure 5) compared with the BCR-ABL fusion gene-expressing CML cell line K562 (0.5 *μ*M) (data are not shown), consistent with previous studies [13]. Imatinib targets BCR-ABL mainly in the cytoplasm via interaction close to the ATP binding site and thus minimally affects ABL expression in the nucleus [14]. Therefore, ABL kinase inhibitors may restrict MM proliferation weakly compared with CML cells because of different underlying mechanisms of tumor cell development. However, imatinib may have synergistic effects in combination therapy regimens for MM.

## 4. Discussion

MM is a genetically heterogeneous disease with diverse clinical outcomes. Increasingly, genetic abnormalities are being explored in MM research and diagnostics because of advances in DNA techniques. Copy number alterations, including whole chromosome and subchromosomal gains and losses, are common contributors to pathogenesis and have demonstrated prognostic impact in MM [15]. In addition, profiling using SNP arrays and next-generation sequencing have extensively and deeply characterized the genomic landscape of MM. FISH, a publicly available resource enabling versatile studies of genome architecture, is a powerful method to study chromosomal organization in single cells. FISH is reliable and provides comprehensive profiling of disease-related genetic aberrations, including deletions, amplifications, inversions, and fusion genes, with a short turn-around time. Thus, FISH could represent a valuable addition to the diagnostic methods currently used for genetic characterization of MM, especially for risk stratification in prognosis and in patients being treated with bortezomib.

The BCR-ABL fusion gene is involved in the Philadelphia chromosome of CML, but it is rarely reported in myelomas. Previous studies have confirmed overexpression of the ABL gene in malignant vs. normal plasma cells [3]. Here, we showed that ABL gene amplification was pervasive in MM patients and MM cell lines by FISH. In addition, ABL protein was easily detectable in MM and other tumor cell lines. ABL gene amplification was more common in MM cells with hyperdiploid karyotypes, which was often accompanied by complex genetic abnormalities, including c-Myc amplification, IGH rearrangement, p53 deletion, and 1q21 amplification. Consistent with previous research [13], our results suggest that tyrosine kinase inhibitors were less efficacious in ABL-amplified MM cells compared with BCR-ABL positive cells. Although the ABL kinase inhibitor imatinib had weak cytotoxicity against MM cells, it may be useful as a component of combination therapy regimens.

This is the first investigation of the role of ABL amplification in MM. We confirmed nuclear ABL gene amplification in MM patients. However, it is regrettable that we were unable to further purify cells using CD138 magnetic beads, which might have better validated chromosomal abnormalities. A future study will be based on CD138 expression immunophenotyping enrichment to assess genotypic differences. ABL expression is prevalent in MM cells in response to DNA damage, and thus, we found no associations between ABL amplification and clinical manifestation or survival. MM is characterized by a high degree of genetic heterogeneity, and almost all patients with MM have chromosomal abnormalities. Currently, there are only a few confirmed chromosomal abnormalities associated with prognostic risk stratification, and most of them are still being explored. ABL amplification was common in hyperdiploid patients (presenting with trisomy characteristics) and was often together with other chromosomal abnormalities so that thousands of abnormal genes expression were involved in a single patient. Therefore, we believe that ABL gene amplification is only a part of the complex chromosomal abnormality of MM and may play a comparative weak role in prognosis. This result remains to be confirmed in larger studies.

Most tumor cells have constitutive ABL activation. However, tyrosine kinase inhibitors have cytotoxic effects in only a few hematologic malignancies with BCR-ABL fusion genes including CML, myelodysplastic syndrome [16], and BCR-ABL-positive MM [17]. However, the combination of tyrosine kinase inhibitors with anti-interleukin-6 antibodies induced marked inhibition of myeloma cell proliferation at low concentrations [3]. In MM and in other hematologic and solid malignancies, genomic instability, centrosome amplification, and aneuploidy are associated with the overexpression of Aurora kinases, a family of serine/threonine kinases that play essential roles in mitosis [18]. Thus, combined inhibition of Aurora and ABL kinases resulted in cell death and tumor regression in MMs mediated by an NF-*κ*B-inducing kinase-c-ABL-STAT3 signaling-centered feedback loop [6]. Conversely, Cai et al. indicated that the activation of c-ABL kinase potentiated the antimyeloma drug lenalidomide by promoting DDA1 recruitment to the CRL4 ubiquitin ligase. Furthermore, panobinostat (a histone deacetylase inhibitor) and dexamethasone enhance lenalidomide-induced substrate degradation and cytotoxicity by activating c-ABL, suggesting a mechanism underlying their synergistic effects with lenalidomide in treating MM [7]. In the past decade, three- or four-drug regimens have become increasingly popular for the treatment of MM. ABL kinase inhibitors can also be applied in these regimens. ABL includes nuclear localization signals and a DNA binding domain that mediate its DNA damage-repair functions. Cytoplasmic ABL has a binding capacity for actin and microtubules to enhance its cytoskeletal remodeling functions. Various types of posttranslational modifications can regulate the catalytic activity, subcellular localization, and stability of ABL, thus affecting cytoplasmic and nuclear ABL functions [19]. Therefore, the regulatory mechanisms of ABL need to be explored to provide a basis for the clinical application of related drugs against hematopoietic malignancies.

## Figures and Tables

**Table 1:** Baseline characteristics of patients with multiple myeloma.

Patient characteristics	ABL amplification (*n* = 67) No. (%)	Normal ABL gene (*n* = 34) No. (%)	*P*
Gender			
Male	48 (71.6)	20 (58.8)	0.194
Female	19 (28.4)	14 (41.2)	

Age (years)			
<**6**	36 (53.7)	21 (61.8)	0.292
≥**65**	31 (46.3)	13 (38.2)	
Median	64	60	

M component			
IgG	44 (65.7)	18 (52.9)	0.133
IgA	20 (29.9)	9 (26.5)	
IgD	0	0	
*λ*	1 (1.5)	3 (8.8)	
*κ*	2 (3.0)	4 (11.8)	

DS stage			
I	7 (10.4)	1 (2.9)	0.476
II	4 (6.0)	2 (5.9)	
III	56 (83.6)	31 (91.2)	

Creatinine (*μ*mol/L)			
≤**177**	55 (82.1)	33 (97.1)	0.055
>**177**	12 (17.9)	1 (2.9)	

Albumin (g/L)			
≥**35**	5 (7.5)	7 (20.6)	0.099
<**35**	62 (92.5)	27 (79.4)	

*β*_2_-MG (mg/L)			
<**3.5**	19 (28.4)	16 (47.1)	0.228
3.5-5.5	26 (38.8)	9 (26.5)	
≥**5.5**	22 (32.8)	9 (26.5)	

LDH (U/L)			
<**250**	49 (73.1)	27 (79.4)	0.475
≥**250**	18 (26.9)	7 (20.6)	

Karyotype			
Hyperdiploid	22 (32.8)	2 (5.9)	<0.001
Hypodiploid	0	6 (17.6)	
Polyploidy	1 (1.50)	0	
Normal karyotype	44 (65.7)	26 (76.5)	

Coexistent adverse cytogenetics			
c-Myc amplification	31 (46.3)	10 (29.4)	0.103
IGH rearrangement	45 (67.2)	20 (58.8)	0.408
D13S319/RB1 deletion	38 (56.7)	22 (64.7)	0.528
*p*53 deletion	5 (14.9)	2 (5.9)	0.768
1*q*21 amplification	34 (50.7)	15 (44.1)	0.440

**Table 2:** Genomic profiling of multiple myeloma cell lines by FISH.

Karyotype	NCI-H929	LP-1	U266	K562
BCR/ABL	2G2O	3G3O = 70%/4G4O = 30%	4G4O = 25%	Multiple F
c-Myc	3F	6F	4F = 27%	
IGH	3F	2F6G2O	2F2O = 21%, FO = 79%	
D13S319/RB1	3G3O	2G2O	2G2O = 19%, GO = 81%	
*p*53/1*q*21	2G3O	3G8-10 O	2G6O = 26%, G2O = 74%	

F: fusion; G: green; O: orange; 2G2O: normal.

## Data Availability

The underlying data used to support our findings of this study are available from the corresponding author on request.
